# A second monoclinic polymorph of *N*-(diethyl­amino­thio­carbon­yl)-*N*′-phenyl­benzamidine

**DOI:** 10.1107/S1600536811010464

**Published:** 2011-03-26

**Authors:** Bernd Schröder, Ligia R. Gomes, Luís M. N. B. F. Santos, Paula Brandão, John Nicolson Low

**Affiliations:** a CICECO, Departamento de Química, Faculdade de Ciências, Universidade do Aveiro, 3810-193 Aveiro, Portugal; b REQUIMTE, Departamento de Química e Bioquímica, Faculdade de Ciências, Universidade do Porto, Rua do Campo Alegre, 687, P-4169_007 Porto, Portugal; c Centro de Investigação em Química, Departamento de Química e Bioquímica, Faculdade de Ciências, Universidade do Porto, Rua do Campo Alegre 687, P-4169_007 Porto, Portugal; d Department of Chemistry, University of Aberdeen, Meston Walk, Old Aberdeen AB24 3UE, Scotland

## Abstract

The asymmetric unit of the title compound, C_18_H_21_N_3_S, contains five mol­ecules. The equivalent bond distances in the five mol­ecules are in excellent agreement, the r.m.s. fit being within 0.007 Å. The five mol­ecules are linked into a chain consisting of alternating pseudo-enanti­omers by N—H⋯S hydrogen bonds supplemented by weak C—H⋯π inter­actions. The action of a glide plane links the asymmetric unit into an extended chain. A polymorph of the title compound with one mol­ecule in the asymmetric unit was reported by Braun *et al.* [*Cryst. Res. Technol.* (1988), **23**, 35–39].

## Related literature

For the structure of the first polymorph, see: Braun *et al.* (1988 ▶). For graph-set analysis, see: Bernstein *et al.* (1995 ▶). For a description of the Cambridge Structural Database, see: Allen (2002 ▶). Preparative details can be found in Beyer *et al.* (1984 ▶).
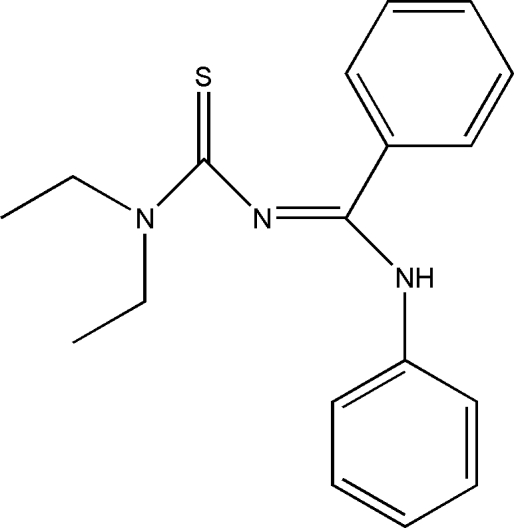


## Experimental

### 

#### Crystal data

C_18_H_21_N_3_S
*M* *_r_* = 311.44
Monoclinic, 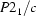

*a* = 21.2894 (8) Å
*b* = 18.8015 (7) Å
*c* = 27.1792 (10) Å
β = 128.584 (2)°
*V* = 8504.1 (5) Å^3^
*Z* = 20
Mo *K*α radiation
μ = 0.19 mm^−1^
*T* = 150 K
0.20 × 0.06 × 0.03 mm

#### Data collection

Bruker SMART APEX diffractometer
Absorption correction: multi-scan (*SADABS*; Bruker, 2004 ▶) *T*_min_ = 0.963, *T*_max_ = 0.994
54002 measured reflections
17402 independent reflections
9835 reflections with *I* > 2σ(*I*)
*R*_int_ = 0.053

#### Refinement

*R*[*F* ^2^ > 2σ(*F* ^2^)] = 0.056
*wR*(*F* ^2^) = 0.142
*S* = 1.01
17398 reflections
1001 parameters
H-atom parameters constrained
Δρ_max_ = 0.34 e Å^−3^
Δρ_min_ = −0.29 e Å^−3^

### 

Data collection: *APEX2* and *SMART* (Bruker, 2004 ▶); cell refinement: *APEX2* and *SAINT* (Bruker, 2004 ▶); data reduction: *SAINT* (Bruker, 2004 ▶); program(s) used to solve structure: *SHELXS97* (Sheldrick, 2008 ▶); program(s) used to refine structure: *SHELXL97* (Sheldrick, 2008 ▶) and *OSCAIL* (McArdle *et al.*, 2004 ▶); molecular graphics: *PLATON* (Spek, 2009 ▶); software used to prepare material for publication: *SHELXL97*.

## Supplementary Material

Crystal structure: contains datablocks global, I. DOI: 10.1107/S1600536811010464/om2413sup1.cif

Structure factors: contains datablocks I. DOI: 10.1107/S1600536811010464/om2413Isup2.hkl

Additional supplementary materials:  crystallographic information; 3D view; checkCIF report

## Figures and Tables

**Table 1:** Hydrogen-bond geometry (Å, °) *Cg*2, *Cg*4, *Cg*5, *Cg*8 and *Cg*10 are the centroids of the C121–C126, C221–C226, C321–C326, C421–C426 and C521–C526 rings, respectively.

*D*—H⋯*A*	*D*—H	H⋯*A*	*D*⋯*A*	*D*—H⋯*A*
N11—H11⋯S24	0.86	2.62	3.440 (2)	159
N21—H21⋯S34	0.86	2.60	3.407 (2)	157
N31—H31⋯S44	0.86	2.64	3.459 (2)	161
N41—H41⋯S54	0.86	2.62	3.416 (2)	155
N51—H51⋯S14^i^	0.86	2.62	3.431 (2)	157
C26—H26*B*⋯*Cg*2	0.99	2.91	3.594 (4)	127
C36—H36*A*⋯*Cg*4	0.99	2.86	3.575 (4)	130
C46—H46*B*⋯*Cg*6	0.99	2.84	3.549 (4)	129
C56—H56*A*⋯*Cg*8	0.99	2.89	3.566 (4)	126
C16—H16*A*⋯*Cg*10^ii^	0.99	2.86	3.552 (4)	128

Symmetry codes: (i) 
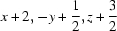
; (ii) 
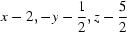
.

**Fig. 1.**
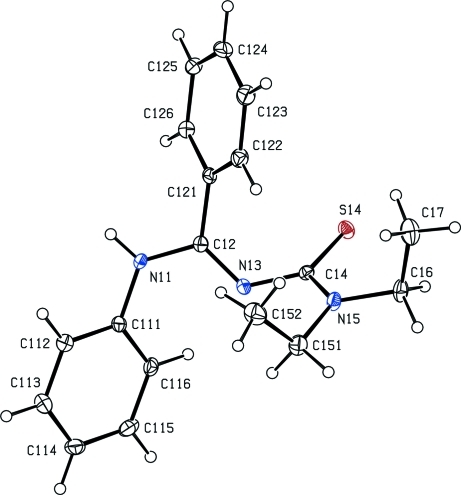
A view of molecule 1 with our numbering scheme. Displacement ellipsoids are drawn at the 30% probability level.

**Fig. 2.**
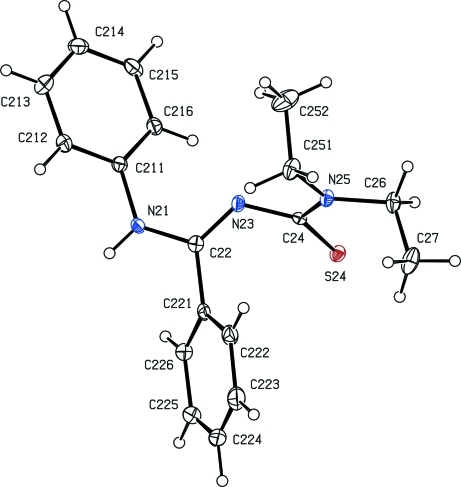
A view of molecule 2 with our numbering scheme. Displacement ellipsoids are drawn at the 30% probability level.

**Fig. 3.**
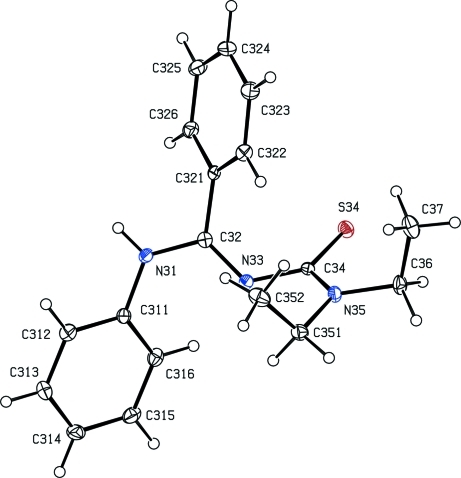
A view of molecule 3 with our numbering scheme. Displacement ellipsoids are drawn at the 30% probability level.

**Fig. 4.**
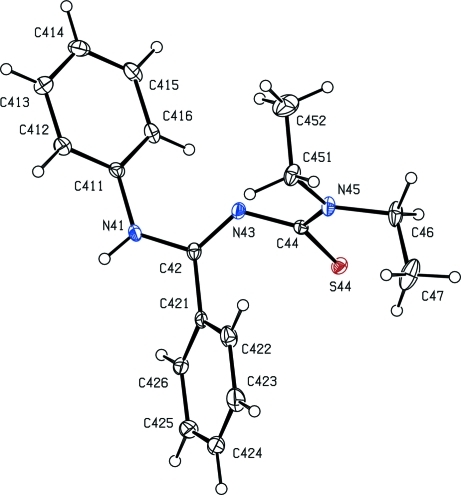
A view of molecule 4 with our numbering scheme. Displacement ellipsoids are drawn at the 30% probability level.

**Fig. 5.**
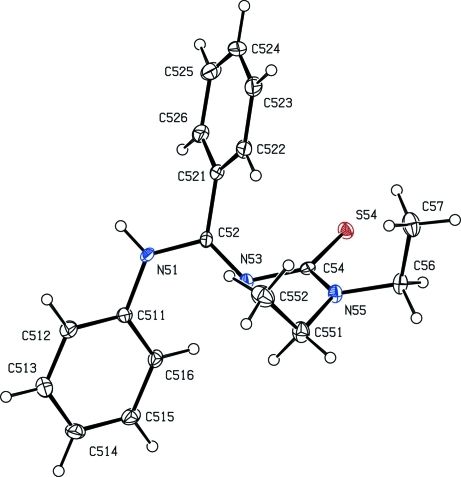
A view of molecule 5 with our numbering scheme. Displacement ellipsoids are drawn at the 30% probability level.

**Fig. 6.**
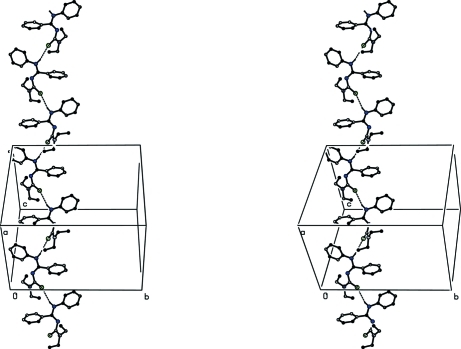
A steroscopic view of the chain formed by the N–H···S hydrogen bonds. Hydrogen atoms not involved in the hydrogen bonding are omitted for the sake of clarity.

## supplementary crystallographic information

### Comment

As part of an investigation into the thermochemical properties of benzamidines a search of the CSD, (Allen, 2002) produced the structural details of C_18_H_21_N_3_S with the code GIDHEG which crystallized in *P*2_1_/*n* with one molecule in the asymmetric unit. This structure was published by Braun *et al.*, 1988, but data acquisition was made at room temperature and it was found to have an ethyl group with urealistic bond lengths and hydrogen atoms on the terminal methyl group were missing. It was thus decided to carry out a new structural determination at 150 K to locate this ethyl group more accurately in order to allow the comparison of the solid state structure with that obtained from gas phase *ab initio* calculations. The low temperature structure crystallizes in *P*2_1_/*c* with cell volume of 8504.1 (5) Å^3^ close to five times that for GIDHEG, 1754.0 (5) Å^3^ and with five molecules in the asymmetric unit. The five independent molecules are shown in Figures 1 - 5.

Equivalent bond distances among the five molecules are in excellent agreement. The r.m.s. fit is within 0.007 Å (Spek, 2009). The five molecules in the asymmetric unit are linked into a chain by N—H···S hydrogen bonds, this chain is supplemented by the action of weak C—H···π interactions. Action of a glide plane links the asymmetric unit into an extended C6 chain, Bernstein *et al.*, 1995, Figure 6.

Figures 1 - 5 show that molecules 1, 3, and 5 are alike and molecules 2 and 4 are alike. The r.m.s. fits of their orthogonal coordinates are obtained from PLATON (Spek, 2003). These pairwise r.m.s. fits are for molecules 1 and 3, 0.202 Å; for molecules 1 and 5, 0.178 Å; for molecules 3 and 5, 0.121 Å and for molecules 2 and 4, 0.142 Å. By contrast, the pairwise r.m.s. fits between molecules in sets 1,3,5 and 2,4 range from 3.808 to 3.900 Å. The only significant differences between the sets are in the conformation of the ethyl group labeled Cx51—Cx52 and in the rotational disposition of H atoms in some of the methyl groups. When molecules 2 and 4 are inverted they map onto molecules 1, 3, and 5 except for the above-mentioned ethyl group. Thus the chain is one of alternating pseudo-enantiomers.

### Experimental

The synthesis of the title compound was performed as described in detail elsewhere, Beyer *et al.* (1984). Pure*N*-(diethylaminothiocarbonyl)benzimide chloride (available through the reaction of equimolar amounts of bis(*N*^'^,*N*^'^-diethyl-*N*-benzoylthioureato) nickel (II) and thionyl chloride in dried THF) reacts with ammonia or primary or secondary amines, in acetone. In the presence of proton trapping reagents (excess of ammonia or an equimolar amount on triethylamine) and after removing of the precipitated triethylamine hydrochloride and successive recrystallization from ethanol, the respective *N*-thiocarbamoylbenzamidine was yielded as pale yellow crystals.

### Refinement

Molecule (1) crystallized in the monoclinic system; space group *P*2_1_/*c* with five N—H···S hydrogen bonded molecules in the asymmetric unit. A consequence of this is that the centre-of-gravity of residues 4 and 5 do not lie within the unit cell. H atoms were treated as riding atoms with C—H(aromatic), 0.95 Å, C—H2(alphatic), 0.99 Å, with *U*_iso_ = 1.2Ueq(C) and C—H3 (methyl), 0.98 Å, with *U*_iso_ = 1.5Ueq(C), H atoms attached to N atoms were located on a difference map, the bond length was initially fixed to 0.860 (1)Å then in the final cycles of refinement allowed to ride on the parent atom with *U*_iso_ = 1.2Ueq(N).

Low angle reflections obscured or partially obscured by the beamstop were omitted.

### Crystal data

**Table:**

C_18_H_21_N_3_S	*F*(000) = 3320
*M**_r_* = 311.44	*D*_x_ = 1.216 Mg m^−^^3^
Monoclinic, *P*2_1_/*c*	Mo *K*α radiation, λ = 0.71073 Å
*a* = 21.2894 (8) Å	Cell parameters from 558 reflections
*b* = 18.8015 (7) Å	θ = 3.5–26.3°
*c* = 27.1792 (10) Å	µ = 0.19 mm^−^^1^
β = 128.584 (2)°	*T* = 150 K
*V* = 8504.1 (5) Å^3^	Plate, yellow
*Z* = 20	0.20 × 0.06 × 0.03 mm

### Data collection

**Table:**

Bruker SMART APEX diffractometer	17402 independent reflections
Radiation source: fine-focus sealed tube	9835 reflections with *I* > 2σ(*I*)
graphite	*R*_int_ = 0.053
Detector resolution: 8.33 pixels mm^-1^	θ_max_ = 26.4°, θ_min_ = 1.2°
ω scans	*h* = −26→26
Absorption correction: multi-scan (*SADABS*; Bruker, 2004)	*k* = −19→23
*T*_min_ = 0.963, *T*_max_ = 0.994	*l* = −33→34
54002 measured reflections	

### Refinement

**Table:**

Refinement on *F*^2^	Primary atom site location: structure-invariant direct methods
Least-squares matrix: full	Secondary atom site location: difference Fourier map
*R*[*F*^2^ > 2σ(*F*^2^)] = 0.056	Hydrogen site location: inferred from neighbouring sites
*wR*(*F*^2^) = 0.142	H-atom parameters constrained
*S* = 1.01	*w* = 1/[σ^2^(*F*_o_^2^) + (0.0532*P*)^2^ + 1.4021*P*] where *P* = (*F*_o_^2^ + 2*F*_c_^2^)/3
17398 reflections	(Δ/σ)_max_ = 0.002
1001 parameters	Δρ_max_ = 0.34 e Å^−^^3^
0 restraints	Δρ_min_ = −0.28 e Å^−^^3^

### Special details

**Table:**

Experimental. Data for C_18_H_21_N_3_S: found 10^2^ w(C)=69.24, 10^2^ w(H)=6.56, 10^2^ w(N)=13.60, 10^2^ w(S)=10.58, calculated 10^2^ w(C)=69.42, 10^2^ w(H)=6.80, 10^2^ w(N)=13.49, 10^2^ w(S)=10.29
Geometry. All e.s.d.'s (except the e.s.d. in the dihedral angle between two l.s. planes) are estimated using the full covariance matrix. The cell e.s.d.'s are taken into account individually in the estimation of e.s.d.'s in distances, angles and torsion angles; correlations between e.s.d.'s in cell parameters are only used when they are defined by crystal symmetry. An approximate (isotropic) treatment of cell e.s.d.'s is used for estimating e.s.d.'s involving l.s. planes.
Refinement. Refinement of *F*^2^ against ALL reflections. The weighted *R*-factor *wR* and goodness of fit *S* are based on *F*^2^, conventional *R*-factors *R* are based on *F*, with *F* set to zero for negative *F*^2^. The threshold expression of *F*^2^ > σ(*F*^2^) is used only for calculating *R*-factors(gt) *etc*. and is not relevant to the choice of reflections for refinement. *R*-factors based on *F*^2^ are statistically about twice as large as those based on *F*, and *R*- factors based on ALL data will be even larger.

### Fractional atomic coordinates and isotropic or equivalent isotropic displacement parameters (Å^2^)

**Table:**

	*x*	*y*	*z*	*U*_iso_*/*U*_eq_	
S14	−0.23152 (4)	0.22025 (3)	0.17728 (3)	0.02237 (16)	
N11	0.02123 (12)	0.17913 (10)	0.34861 (10)	0.0198 (5)	
H11	0.0596	0.2088	0.3727	0.024*	
N13	−0.09015 (12)	0.15087 (10)	0.24855 (10)	0.0205 (5)	
N15	−0.16587 (12)	0.13186 (10)	0.14303 (10)	0.0219 (5)	
C12	−0.04052 (15)	0.19866 (12)	0.28844 (12)	0.0175 (6)	
C14	−0.15869 (15)	0.16731 (11)	0.18921 (12)	0.0180 (6)	
C16	−0.23483 (16)	0.14413 (14)	0.07668 (13)	0.0280 (6)	
H16A	−0.2836	0.1520	0.0730	0.034*	
H16B	−0.2438	0.1013	0.0519	0.034*	
C17	−0.22199 (17)	0.20757 (15)	0.04975 (14)	0.0364 (7)	
H17A	−0.2682	0.2128	0.0051	0.055*	
H17B	−0.1731	0.2005	0.0541	0.055*	
H17C	−0.2163	0.2506	0.0726	0.055*	
C111	0.03899 (16)	0.10834 (12)	0.37206 (12)	0.0200 (6)	
C112	0.11730 (16)	0.08396 (13)	0.40673 (13)	0.0268 (6)	
H112	0.1582	0.1147	0.4148	0.032*	
C113	0.13615 (17)	0.01545 (13)	0.42952 (13)	0.0327 (7)	
H113	0.1900	−0.0008	0.4530	0.039*	
C114	0.07749 (18)	−0.03008 (13)	0.41861 (14)	0.0322 (7)	
H114	0.0906	−0.0774	0.4342	0.039*	
C115	−0.00069 (18)	−0.00528 (13)	0.38444 (13)	0.0303 (7)	
H115	−0.0413	−0.0359	0.3769	0.036*	
C116	−0.02042 (16)	0.06343 (12)	0.36115 (12)	0.0229 (6)	
H116	−0.0742	0.0798	0.3378	0.028*	
C121	−0.03988 (15)	0.27481 (12)	0.27319 (12)	0.0195 (6)	
C122	−0.04437 (16)	0.29136 (13)	0.22116 (13)	0.0263 (6)	
H122	−0.0503	0.2544	0.1947	0.032*	
C123	−0.04029 (16)	0.36125 (13)	0.20777 (13)	0.0316 (7)	
H123	−0.0427	0.3721	0.1725	0.038*	
C124	−0.03274 (16)	0.41535 (14)	0.24531 (14)	0.0332 (7)	
H124	−0.0301	0.4634	0.2358	0.040*	
C125	−0.02897 (16)	0.39982 (13)	0.29668 (13)	0.0287 (7)	
H125	−0.0242	0.4372	0.3223	0.034*	
C126	−0.03208 (15)	0.32944 (12)	0.31115 (13)	0.0231 (6)	
H126	−0.0289	0.3188	0.3468	0.028*	
C151	−0.10461 (15)	0.07888 (13)	0.15722 (13)	0.0291 (7)	
H15A	−0.1290	0.0474	0.1202	0.035*	
H15B	−0.0904	0.0490	0.1929	0.035*	
C152	−0.02819 (16)	0.11010 (14)	0.17348 (14)	0.0363 (7)	
H15C	0.0054	0.0718	0.1767	0.054*	
H15D	0.0012	0.1352	0.2138	0.054*	
H15E	−0.0417	0.1435	0.1405	0.054*	
S24	0.17634 (4)	0.27346 (3)	0.47670 (3)	0.02079 (16)	
N21	0.42915 (12)	0.30005 (10)	0.64607 (10)	0.0211 (5)	
H21	0.4629	0.2678	0.6713	0.025*	
N23	0.32595 (12)	0.32552 (10)	0.54203 (10)	0.0197 (5)	
N25	0.25155 (12)	0.32914 (10)	0.43525 (10)	0.0198 (5)	
C22	0.36825 (15)	0.27907 (12)	0.58633 (13)	0.0194 (6)	
C24	0.25506 (15)	0.30905 (11)	0.48411 (12)	0.0168 (5)	
C26	0.17791 (15)	0.32437 (13)	0.37021 (12)	0.0261 (6)	
H26A	0.1759	0.3646	0.3458	0.031*	
H26B	0.1309	0.3282	0.3694	0.031*	
C27	0.17323 (16)	0.25506 (14)	0.33968 (13)	0.0359 (7)	
H27A	0.1251	0.2551	0.2954	0.054*	
H27B	0.1705	0.2152	0.3616	0.054*	
H27C	0.2211	0.2500	0.3423	0.054*	
C211	0.44753 (15)	0.37149 (12)	0.66763 (12)	0.0201 (6)	
C212	0.52721 (16)	0.39308 (13)	0.70694 (13)	0.0279 (7)	
H212	0.5683	0.3609	0.7175	0.033*	
C213	0.54674 (18)	0.46140 (14)	0.73072 (14)	0.0359 (7)	
H213	0.6014	0.4760	0.7575	0.043*	
C214	0.48745 (19)	0.50886 (13)	0.71601 (14)	0.0326 (7)	
H214	0.5011	0.5558	0.7325	0.039*	
C215	0.40839 (18)	0.48707 (13)	0.67718 (13)	0.0301 (7)	
H215	0.3676	0.5194	0.6671	0.036*	
C216	0.38750 (16)	0.41883 (12)	0.65263 (12)	0.0244 (6)	
H216	0.3328	0.4044	0.6258	0.029*	
C221	0.35905 (15)	0.20031 (12)	0.57676 (13)	0.0233 (6)	
C222	0.35586 (16)	0.16999 (14)	0.52872 (13)	0.0319 (7)	
H222	0.3583	0.1993	0.5015	0.038*	
C223	0.34902 (18)	0.09671 (16)	0.52042 (15)	0.0460 (9)	
H223	0.3475	0.0758	0.4879	0.055*	
C224	0.34440 (18)	0.05451 (15)	0.55948 (16)	0.0494 (10)	
H224	0.3391	0.0045	0.5534	0.059*	
C225	0.34739 (17)	0.08392 (14)	0.60681 (15)	0.0422 (8)	
H225	0.3439	0.0542	0.6333	0.051*	
C226	0.35553 (16)	0.15713 (13)	0.61652 (14)	0.0303 (7)	
H226	0.3587	0.1774	0.6500	0.036*	
C251	0.32204 (16)	0.35829 (13)	0.44386 (13)	0.0283 (6)	
H25A	0.3182	0.3467	0.4065	0.034*	
H25B	0.3714	0.3357	0.4810	0.034*	
C252	0.32810 (19)	0.43781 (14)	0.45297 (16)	0.0462 (9)	
H25C	0.3723	0.4560	0.4544	0.069*	
H25D	0.3383	0.4491	0.4926	0.069*	
H25E	0.2776	0.4600	0.4179	0.069*	
S34	0.57027 (4)	0.20604 (3)	0.77842 (3)	0.02182 (16)	
N31	0.82249 (12)	0.16993 (9)	0.94887 (10)	0.0199 (5)	
H31	0.8584	0.2014	0.9731	0.024*	
N33	0.71349 (12)	0.13984 (9)	0.84771 (10)	0.0187 (5)	
N35	0.63691 (12)	0.12304 (10)	0.74181 (10)	0.0190 (5)	
C32	0.76082 (15)	0.18831 (12)	0.88835 (12)	0.0175 (6)	
C34	0.64369 (15)	0.15580 (11)	0.78852 (12)	0.0164 (5)	
C36	0.56655 (15)	0.13427 (13)	0.67604 (12)	0.0240 (6)	
H36A	0.5184	0.1409	0.6735	0.029*	
H36B	0.5578	0.0915	0.6512	0.029*	
C37	0.57675 (16)	0.19867 (14)	0.64800 (13)	0.0319 (7)	
H37A	0.5291	0.2040	0.6039	0.048*	
H37B	0.6243	0.1923	0.6504	0.048*	
H37C	0.5835	0.2413	0.6716	0.048*	
C311	0.84020 (15)	0.10053 (12)	0.97507 (12)	0.0186 (6)	
C312	0.91956 (16)	0.07851 (13)	1.01483 (13)	0.0274 (6)	
H312	0.9607	0.1091	1.0233	0.033*	
C313	0.93886 (17)	0.01171 (13)	1.04232 (13)	0.0331 (7)	
H313	0.9934	−0.0032	1.0696	0.040*	
C314	0.87961 (18)	−0.03343 (13)	1.03053 (13)	0.0314 (7)	
H314	0.8930	−0.0792	1.0493	0.038*	
C315	0.80036 (17)	−0.01083 (13)	0.99084 (13)	0.0268 (6)	
H315	0.7592	−0.0416	0.9823	0.032*	
C316	0.78034 (16)	0.05582 (12)	0.96349 (12)	0.0228 (6)	
H316	0.7259	0.0710	0.9369	0.027*	
C321	0.75888 (14)	0.26532 (12)	0.87418 (12)	0.0184 (6)	
C322	0.75293 (15)	0.28526 (12)	0.82203 (13)	0.0247 (6)	
H322	0.7472	0.2499	0.7945	0.030*	
C323	0.75536 (16)	0.35619 (13)	0.81015 (14)	0.0317 (7)	
H323	0.7519	0.3695	0.7748	0.038*	
C324	0.76285 (17)	0.40776 (13)	0.84963 (14)	0.0331 (7)	
H324	0.7644	0.4565	0.8413	0.040*	
C325	0.76810 (16)	0.38887 (13)	0.90082 (14)	0.0298 (7)	
H325	0.7727	0.4247	0.9275	0.036*	
C326	0.76675 (15)	0.31776 (12)	0.91388 (13)	0.0232 (6)	
H326	0.7712	0.3049	0.9497	0.028*	
C351	0.70025 (15)	0.07513 (12)	0.75451 (13)	0.0235 (6)	
H35A	0.6769	0.0444	0.7172	0.028*	
H35B	0.7173	0.0438	0.7902	0.028*	
C352	0.77462 (16)	0.11171 (13)	0.76987 (14)	0.0303 (7)	
H35C	0.8092	0.0763	0.7709	0.045*	
H35D	0.8043	0.1349	0.8110	0.045*	
H35E	0.7582	0.1474	0.7376	0.045*	
S44	0.97103 (4)	0.27165 (3)	1.07600 (3)	0.02064 (16)	
N41	1.22665 (12)	0.30294 (10)	1.24426 (10)	0.0205 (5)	
H41	1.2641	0.2729	1.2689	0.025*	
N43	1.11834 (13)	0.32832 (10)	1.14204 (10)	0.0199 (5)	
N45	1.04558 (12)	0.33060 (10)	1.03541 (10)	0.0213 (5)	
C42	1.16571 (15)	0.28189 (12)	1.18444 (12)	0.0186 (6)	
C44	1.04862 (15)	0.31032 (11)	1.08374 (12)	0.0179 (5)	
C46	0.97333 (16)	0.32224 (14)	0.97023 (13)	0.0310 (7)	
H46A	0.9704	0.3615	0.9446	0.037*	
H46B	0.9254	0.3252	0.9683	0.037*	
C47	0.97268 (17)	0.25186 (16)	0.94288 (14)	0.0434 (8)	
H47A	0.9247	0.2491	0.8986	0.065*	
H47B	0.9720	0.2128	0.9664	0.065*	
H47C	1.0209	0.2481	0.9459	0.065*	
C411	1.24352 (15)	0.37451 (12)	1.26575 (12)	0.0197 (6)	
C412	1.32171 (16)	0.39872 (13)	1.30205 (13)	0.0280 (7)	
H412	1.3634	0.3677	1.3119	0.034*	
C413	1.33974 (18)	0.46792 (13)	1.32419 (14)	0.0344 (7)	
H413	1.3937	0.4843	1.3492	0.041*	
C414	1.27925 (19)	0.51327 (13)	1.30987 (14)	0.0352 (7)	
H414	1.2915	0.5610	1.3244	0.042*	
C415	1.20113 (18)	0.48899 (13)	1.27444 (13)	0.0297 (7)	
H415	1.1598	0.5199	1.2653	0.036*	
C416	1.18272 (16)	0.41976 (13)	1.25222 (12)	0.0244 (6)	
H416	1.1289	0.4032	1.2279	0.029*	
C421	1.16223 (15)	0.20419 (12)	1.17216 (12)	0.0202 (6)	
C422	1.15931 (16)	0.18123 (14)	1.12229 (13)	0.0304 (7)	
H422	1.1595	0.2151	1.0964	0.036*	
C423	1.15607 (17)	0.10929 (15)	1.10993 (14)	0.0390 (8)	
H423	1.1547	0.0938	1.0760	0.047*	
C424	1.15479 (16)	0.06015 (14)	1.14698 (15)	0.0376 (8)	
H424	1.1519	0.0108	1.1382	0.045*	
C425	1.15765 (16)	0.08220 (13)	1.19641 (14)	0.0322 (7)	
H425	1.1569	0.0481	1.2218	0.039*	
C426	1.16169 (15)	0.15433 (12)	1.20946 (13)	0.0239 (6)	
H426	1.1641	0.1694	1.2440	0.029*	
C451	1.11588 (15)	0.36182 (13)	1.04462 (13)	0.0272 (6)	
H45A	1.1124	0.3516	1.0072	0.033*	
H45B	1.1655	0.3393	1.0816	0.033*	
C452	1.12087 (19)	0.44068 (14)	1.05463 (15)	0.0457 (9)	
H45C	1.1645	0.4601	1.0559	0.069*	
H45D	1.1314	0.4508	1.0946	0.069*	
H45E	1.0699	0.4627	1.0200	0.069*	
S54	1.37598 (4)	0.21402 (3)	1.37768 (3)	0.02200 (16)	
N51	1.62793 (12)	0.18011 (10)	1.54716 (10)	0.0209 (5)	
H51	1.6617	0.2131	1.5711	0.025*	
N53	1.52037 (12)	0.14940 (10)	1.44597 (10)	0.0189 (5)	
N55	1.44223 (12)	0.13268 (10)	1.34005 (10)	0.0211 (5)	
C52	1.56515 (15)	0.19807 (12)	1.48708 (12)	0.0182 (6)	
C54	1.45012 (15)	0.16539 (11)	1.38743 (12)	0.0183 (6)	
C56	1.37164 (16)	0.14475 (13)	1.27431 (12)	0.0270 (6)	
H56A	1.3234	0.1494	1.2718	0.032*	
H56B	1.3638	0.1031	1.2487	0.032*	
C57	1.38034 (17)	0.21090 (14)	1.24744 (13)	0.0334 (7)	
H57A	1.3326	0.2166	1.2034	0.050*	
H57B	1.4281	0.2065	1.2499	0.050*	
H57C	1.3860	0.2525	1.2717	0.050*	
C511	1.64861 (16)	0.11016 (12)	1.57227 (12)	0.0205 (6)	
C512	1.72888 (16)	0.09059 (13)	1.61213 (13)	0.0276 (7)	
H512	1.7688	0.1234	1.6211	0.033*	
C513	1.75108 (18)	0.02355 (14)	1.63886 (14)	0.0334 (7)	
H513	1.8062	0.0104	1.6660	0.040*	
C514	1.69336 (18)	−0.02463 (13)	1.62629 (13)	0.0319 (7)	
H514	1.7086	−0.0708	1.6444	0.038*	
C515	1.61325 (18)	−0.00462 (13)	1.58699 (13)	0.0290 (7)	
H515	1.5734	−0.0372	1.5784	0.035*	
C516	1.59073 (16)	0.06252 (12)	1.56013 (12)	0.0232 (6)	
H516	1.5357	0.0759	1.5334	0.028*	
C521	1.55848 (15)	0.27605 (12)	1.47388 (13)	0.0239 (6)	
C522	1.55626 (16)	0.29979 (13)	1.42436 (13)	0.0319 (7)	
H522	1.5583	0.2667	1.3990	0.038*	
C523	1.55099 (18)	0.37207 (15)	1.41215 (15)	0.0465 (9)	
H523	1.5503	0.3887	1.3788	0.056*	
C524	1.54682 (19)	0.41941 (15)	1.44827 (17)	0.0539 (10)	
H524	1.5427	0.4689	1.4395	0.065*	
C525	1.54845 (18)	0.39633 (14)	1.49707 (17)	0.0474 (9)	
H525	1.5455	0.4298	1.5218	0.057*	
C526	1.55436 (16)	0.32412 (13)	1.51028 (14)	0.0320 (7)	
H526	1.5556	0.3080	1.5440	0.038*	
C551	1.50538 (15)	0.08427 (13)	1.35181 (13)	0.0286 (7)	
H55A	1.4815	0.0534	1.3145	0.034*	
H55B	1.5238	0.0532	1.3879	0.034*	
C552	1.57743 (17)	0.12203 (14)	1.36545 (15)	0.0399 (8)	
H55C	1.6128	0.0872	1.3669	0.060*	
H55D	1.6068	0.1465	1.4061	0.060*	
H55E	1.5594	0.1569	1.3322	0.060*	

### Atomic displacement parameters (Å^2^)

**Table:**

	*U*^11^	*U*^22^	*U*^33^	*U*^12^	*U*^13^	*U*^23^
S14	0.0212 (4)	0.0243 (3)	0.0205 (4)	0.0059 (3)	0.0124 (4)	0.0011 (3)
N11	0.0176 (12)	0.0184 (10)	0.0149 (13)	−0.0041 (9)	0.0059 (11)	−0.0017 (9)
N13	0.0183 (12)	0.0188 (10)	0.0169 (13)	0.0023 (9)	0.0074 (11)	0.0007 (10)
N15	0.0171 (12)	0.0265 (11)	0.0166 (13)	0.0003 (9)	0.0077 (11)	−0.0046 (10)
C12	0.0159 (14)	0.0202 (12)	0.0177 (16)	0.0007 (11)	0.0111 (14)	−0.0005 (11)
C14	0.0177 (14)	0.0168 (12)	0.0181 (15)	−0.0026 (10)	0.0105 (13)	−0.0008 (11)
C16	0.0211 (16)	0.0409 (15)	0.0165 (16)	0.0009 (13)	0.0091 (14)	−0.0053 (13)
C17	0.0312 (18)	0.0553 (19)	0.0253 (19)	0.0032 (14)	0.0189 (17)	0.0048 (15)
C111	0.0236 (15)	0.0195 (12)	0.0125 (15)	0.0005 (11)	0.0091 (14)	0.0002 (11)
C112	0.0229 (16)	0.0241 (13)	0.0268 (18)	−0.0010 (12)	0.0122 (15)	0.0007 (12)
C113	0.0288 (18)	0.0296 (15)	0.0274 (18)	0.0057 (13)	0.0116 (16)	0.0040 (13)
C114	0.044 (2)	0.0189 (13)	0.0251 (18)	−0.0015 (13)	0.0176 (17)	−0.0014 (12)
C115	0.0396 (19)	0.0246 (14)	0.0238 (18)	−0.0133 (13)	0.0183 (16)	−0.0056 (13)
C116	0.0217 (15)	0.0268 (13)	0.0143 (16)	−0.0032 (12)	0.0083 (14)	−0.0015 (12)
C121	0.0133 (14)	0.0208 (12)	0.0199 (16)	−0.0001 (11)	0.0082 (14)	0.0024 (11)
C122	0.0253 (16)	0.0290 (14)	0.0227 (17)	−0.0033 (12)	0.0140 (15)	0.0007 (12)
C123	0.0320 (18)	0.0342 (15)	0.0237 (18)	−0.0069 (13)	0.0149 (16)	0.0052 (13)
C124	0.0271 (17)	0.0225 (14)	0.038 (2)	−0.0017 (12)	0.0145 (17)	0.0085 (14)
C125	0.0220 (16)	0.0214 (13)	0.0303 (18)	−0.0014 (12)	0.0102 (15)	−0.0041 (13)
C126	0.0215 (16)	0.0225 (13)	0.0215 (17)	0.0002 (11)	0.0115 (15)	−0.0002 (12)
C151	0.0262 (16)	0.0313 (14)	0.0275 (18)	0.0025 (12)	0.0156 (15)	−0.0061 (13)
C152	0.0322 (18)	0.0380 (16)	0.045 (2)	0.0072 (14)	0.0268 (18)	0.0022 (15)
S24	0.0193 (4)	0.0225 (3)	0.0207 (4)	−0.0038 (3)	0.0126 (4)	−0.0009 (3)
N21	0.0218 (13)	0.0174 (10)	0.0156 (13)	0.0047 (9)	0.0074 (12)	0.0001 (9)
N23	0.0168 (12)	0.0231 (11)	0.0139 (13)	−0.0022 (9)	0.0070 (11)	0.0000 (10)
N25	0.0173 (12)	0.0248 (11)	0.0146 (13)	−0.0035 (9)	0.0087 (11)	−0.0005 (9)
C22	0.0193 (15)	0.0225 (13)	0.0189 (16)	0.0004 (11)	0.0132 (14)	−0.0009 (12)
C24	0.0166 (14)	0.0119 (11)	0.0188 (15)	0.0024 (10)	0.0095 (13)	0.0016 (11)
C26	0.0193 (15)	0.0385 (15)	0.0154 (16)	−0.0025 (12)	0.0083 (14)	0.0021 (13)
C27	0.0231 (17)	0.0593 (19)	0.0276 (19)	−0.0092 (14)	0.0169 (16)	−0.0143 (15)
C211	0.0233 (15)	0.0198 (12)	0.0157 (15)	0.0010 (11)	0.0115 (14)	0.0003 (11)
C212	0.0199 (16)	0.0263 (14)	0.0270 (18)	0.0044 (12)	0.0095 (15)	0.0010 (13)
C213	0.0301 (18)	0.0314 (15)	0.035 (2)	−0.0094 (14)	0.0146 (17)	−0.0055 (14)
C214	0.047 (2)	0.0205 (13)	0.0246 (18)	0.0003 (14)	0.0193 (17)	0.0002 (13)
C215	0.0395 (19)	0.0251 (14)	0.0212 (17)	0.0133 (13)	0.0167 (16)	0.0046 (13)
C216	0.0236 (16)	0.0247 (13)	0.0185 (16)	0.0046 (12)	0.0101 (14)	0.0011 (12)
C221	0.0149 (15)	0.0226 (13)	0.0198 (16)	0.0048 (11)	0.0046 (14)	−0.0006 (12)
C222	0.0252 (17)	0.0319 (15)	0.0248 (18)	0.0078 (13)	0.0088 (15)	−0.0050 (13)
C223	0.0336 (19)	0.0400 (18)	0.034 (2)	0.0128 (15)	0.0062 (18)	−0.0141 (16)
C224	0.032 (2)	0.0232 (15)	0.046 (2)	0.0049 (14)	0.0011 (19)	−0.0068 (16)
C225	0.0308 (19)	0.0250 (15)	0.041 (2)	−0.0012 (13)	0.0081 (18)	0.0055 (15)
C226	0.0247 (17)	0.0241 (14)	0.0298 (19)	−0.0001 (12)	0.0110 (16)	0.0000 (13)
C251	0.0242 (16)	0.0390 (15)	0.0239 (17)	−0.0081 (13)	0.0161 (15)	−0.0028 (13)
C252	0.059 (2)	0.0419 (17)	0.052 (2)	−0.0242 (16)	0.041 (2)	−0.0113 (16)
S34	0.0202 (4)	0.0231 (3)	0.0210 (4)	0.0039 (3)	0.0122 (4)	0.0009 (3)
N31	0.0212 (13)	0.0172 (10)	0.0153 (13)	−0.0048 (9)	0.0085 (12)	−0.0005 (9)
N33	0.0183 (12)	0.0177 (10)	0.0167 (13)	−0.0019 (9)	0.0092 (11)	0.0002 (9)
N35	0.0163 (12)	0.0209 (10)	0.0155 (13)	0.0027 (9)	0.0078 (11)	−0.0011 (9)
C32	0.0173 (14)	0.0206 (12)	0.0170 (16)	0.0004 (11)	0.0118 (14)	0.0006 (12)
C34	0.0165 (14)	0.0137 (11)	0.0157 (15)	−0.0014 (10)	0.0083 (13)	0.0028 (11)
C36	0.0205 (15)	0.0312 (14)	0.0139 (16)	0.0025 (12)	0.0075 (14)	−0.0021 (12)
C37	0.0288 (18)	0.0429 (16)	0.0234 (18)	0.0079 (13)	0.0160 (16)	0.0072 (14)
C311	0.0215 (15)	0.0188 (12)	0.0126 (15)	−0.0006 (11)	0.0091 (13)	−0.0005 (11)
C312	0.0242 (16)	0.0247 (13)	0.0272 (18)	−0.0040 (12)	0.0130 (15)	0.0001 (13)
C313	0.0274 (17)	0.0285 (14)	0.0237 (18)	0.0051 (13)	0.0063 (15)	0.0040 (13)
C314	0.0432 (19)	0.0193 (13)	0.0196 (17)	−0.0018 (13)	0.0137 (16)	0.0026 (12)
C315	0.0352 (18)	0.0233 (13)	0.0202 (17)	−0.0099 (12)	0.0165 (16)	−0.0030 (12)
C316	0.0235 (16)	0.0279 (14)	0.0172 (16)	−0.0035 (12)	0.0129 (14)	−0.0021 (12)
C321	0.0113 (14)	0.0173 (12)	0.0193 (16)	−0.0023 (10)	0.0060 (13)	0.0013 (11)
C322	0.0230 (16)	0.0234 (13)	0.0242 (17)	−0.0020 (11)	0.0130 (15)	−0.0003 (12)
C323	0.0307 (18)	0.0277 (14)	0.0295 (19)	−0.0055 (13)	0.0153 (16)	0.0059 (14)
C324	0.0306 (18)	0.0191 (13)	0.035 (2)	−0.0044 (12)	0.0136 (17)	0.0054 (13)
C325	0.0222 (16)	0.0196 (13)	0.039 (2)	−0.0023 (12)	0.0150 (16)	−0.0063 (13)
C326	0.0188 (15)	0.0225 (13)	0.0248 (17)	−0.0019 (11)	0.0120 (14)	−0.0019 (12)
C351	0.0252 (16)	0.0201 (12)	0.0235 (17)	0.0041 (11)	0.0143 (15)	−0.0017 (12)
C352	0.0310 (17)	0.0284 (14)	0.041 (2)	0.0085 (12)	0.0270 (17)	0.0058 (13)
S44	0.0200 (4)	0.0220 (3)	0.0196 (4)	−0.0041 (3)	0.0122 (4)	−0.0013 (3)
N41	0.0162 (12)	0.0208 (10)	0.0156 (13)	0.0044 (9)	0.0056 (11)	0.0017 (9)
N43	0.0184 (12)	0.0216 (10)	0.0141 (13)	−0.0030 (9)	0.0074 (11)	−0.0002 (10)
N45	0.0155 (12)	0.0303 (11)	0.0140 (13)	−0.0038 (9)	0.0072 (11)	0.0019 (10)
C42	0.0145 (14)	0.0238 (13)	0.0169 (16)	−0.0010 (11)	0.0095 (14)	0.0010 (12)
C44	0.0178 (14)	0.0153 (11)	0.0169 (15)	0.0021 (11)	0.0090 (13)	−0.0005 (11)
C46	0.0205 (16)	0.0521 (17)	0.0138 (16)	−0.0064 (14)	0.0075 (14)	0.0040 (14)
C47	0.0300 (18)	0.075 (2)	0.031 (2)	−0.0165 (16)	0.0216 (17)	−0.0232 (17)
C411	0.0240 (15)	0.0178 (12)	0.0151 (15)	0.0021 (11)	0.0112 (14)	0.0025 (11)
C412	0.0233 (16)	0.0252 (14)	0.0299 (18)	0.0033 (12)	0.0138 (16)	0.0022 (13)
C413	0.0333 (18)	0.0261 (14)	0.0311 (19)	−0.0056 (13)	0.0139 (17)	−0.0018 (14)
C414	0.051 (2)	0.0197 (13)	0.0280 (19)	0.0029 (14)	0.0208 (18)	0.0025 (13)
C415	0.0372 (19)	0.0280 (14)	0.0201 (17)	0.0145 (13)	0.0161 (16)	0.0051 (13)
C416	0.0218 (16)	0.0302 (14)	0.0180 (16)	0.0077 (12)	0.0107 (14)	0.0040 (12)
C421	0.0122 (14)	0.0224 (13)	0.0166 (16)	0.0033 (11)	0.0044 (13)	−0.0007 (11)
C422	0.0278 (17)	0.0364 (15)	0.0204 (17)	0.0086 (13)	0.0118 (15)	−0.0015 (13)
C423	0.0326 (19)	0.0402 (17)	0.0292 (19)	0.0143 (14)	0.0120 (17)	−0.0085 (15)
C424	0.0236 (17)	0.0244 (14)	0.039 (2)	0.0065 (13)	0.0067 (16)	−0.0087 (14)
C425	0.0241 (17)	0.0242 (14)	0.038 (2)	0.0025 (12)	0.0142 (16)	−0.0002 (14)
C426	0.0178 (15)	0.0232 (13)	0.0229 (17)	−0.0004 (11)	0.0088 (14)	−0.0028 (12)
C451	0.0223 (15)	0.0377 (15)	0.0207 (16)	−0.0086 (12)	0.0129 (14)	−0.0023 (13)
C452	0.052 (2)	0.0405 (17)	0.051 (2)	−0.0165 (16)	0.035 (2)	−0.0024 (16)
S54	0.0187 (4)	0.0230 (3)	0.0223 (4)	0.0039 (3)	0.0118 (4)	0.0011 (3)
N51	0.0211 (13)	0.0168 (10)	0.0164 (13)	−0.0082 (9)	0.0076 (12)	−0.0021 (9)
N53	0.0150 (12)	0.0198 (10)	0.0178 (13)	0.0005 (9)	0.0082 (11)	0.0013 (10)
N55	0.0161 (12)	0.0261 (11)	0.0180 (13)	0.0019 (9)	0.0091 (11)	−0.0013 (10)
C52	0.0138 (14)	0.0216 (12)	0.0144 (15)	−0.0010 (11)	0.0064 (13)	−0.0002 (11)
C54	0.0178 (14)	0.0138 (11)	0.0217 (16)	−0.0035 (10)	0.0115 (14)	0.0003 (11)
C56	0.0198 (15)	0.0365 (15)	0.0182 (16)	−0.0005 (12)	0.0087 (14)	−0.0019 (13)
C57	0.0275 (17)	0.0496 (17)	0.0245 (18)	0.0039 (14)	0.0168 (16)	0.0068 (14)
C511	0.0269 (16)	0.0184 (12)	0.0132 (15)	−0.0018 (11)	0.0111 (14)	0.0003 (11)
C512	0.0251 (17)	0.0255 (14)	0.0277 (18)	−0.0051 (12)	0.0142 (16)	−0.0001 (13)
C513	0.0280 (17)	0.0333 (15)	0.0264 (18)	0.0045 (13)	0.0108 (16)	0.0021 (14)
C514	0.045 (2)	0.0194 (13)	0.0246 (18)	0.0004 (13)	0.0187 (17)	0.0029 (13)
C515	0.0382 (19)	0.0245 (13)	0.0221 (17)	−0.0108 (13)	0.0177 (16)	−0.0006 (12)
C516	0.0229 (15)	0.0269 (13)	0.0150 (15)	−0.0050 (12)	0.0094 (14)	0.0000 (12)
C521	0.0150 (15)	0.0200 (12)	0.0247 (17)	−0.0033 (11)	0.0066 (14)	0.0007 (12)
C522	0.0246 (17)	0.0307 (15)	0.0244 (18)	−0.0071 (13)	0.0074 (15)	0.0056 (13)
C523	0.035 (2)	0.0355 (17)	0.034 (2)	−0.0143 (15)	0.0043 (18)	0.0121 (16)
C524	0.035 (2)	0.0198 (15)	0.054 (3)	−0.0068 (14)	0.002 (2)	0.0091 (17)
C525	0.0291 (19)	0.0245 (15)	0.058 (3)	−0.0017 (14)	0.0123 (19)	−0.0097 (16)
C526	0.0204 (16)	0.0259 (14)	0.035 (2)	−0.0020 (12)	0.0100 (16)	−0.0039 (14)
C551	0.0217 (16)	0.0354 (15)	0.0270 (18)	0.0049 (12)	0.0144 (15)	−0.0019 (13)
C552	0.0364 (19)	0.0389 (16)	0.050 (2)	0.0078 (14)	0.0297 (19)	0.0088 (16)

### Geometric parameters (Å, °)

**Table:**

S14—C14	1.695 (2)	C314—C315	1.386 (4)
N11—C12	1.362 (3)	C314—H314	0.9500
N11—C111	1.421 (3)	C315—C316	1.382 (3)
N11—H11	0.8601	C315—H315	0.9500
N13—C12	1.292 (3)	C316—H316	0.9500
N13—C14	1.375 (3)	C321—C326	1.392 (3)
N15—C14	1.343 (3)	C321—C322	1.394 (3)
N15—C16	1.469 (3)	C322—C323	1.381 (3)
N15—C151	1.488 (3)	C322—H322	0.9500
C12—C121	1.493 (3)	C323—C324	1.380 (4)
C16—C17	1.511 (3)	C323—H323	0.9500
C16—H16A	0.9900	C324—C325	1.371 (4)
C16—H16B	0.9900	C324—H324	0.9500
C17—H17A	0.9800	C325—C326	1.388 (3)
C17—H17B	0.9800	C325—H325	0.9500
C17—H17C	0.9800	C326—H326	0.9500
C111—C112	1.385 (3)	C351—C352	1.526 (3)
C111—C116	1.390 (3)	C351—H35A	0.9900
C112—C113	1.376 (3)	C351—H35B	0.9900
C112—H112	0.9500	C352—H35C	0.9800
C113—C114	1.385 (4)	C352—H35D	0.9800
C113—H113	0.9500	C352—H35E	0.9800
C114—C115	1.386 (4)	S44—C44	1.694 (2)
C114—H114	0.9500	N41—C42	1.361 (3)
C115—C116	1.384 (3)	N41—C411	1.421 (3)
C115—H115	0.9500	N41—H41	0.8592
C116—H116	0.9500	N43—C42	1.287 (3)
C121—C126	1.390 (3)	N43—C44	1.377 (3)
C121—C122	1.392 (3)	N45—C44	1.330 (3)
C122—C123	1.381 (3)	N45—C46	1.460 (3)
C122—H122	0.9500	N45—C451	1.476 (3)
C123—C124	1.377 (4)	C42—C421	1.490 (3)
C123—H123	0.9500	C46—C47	1.513 (4)
C124—C125	1.379 (4)	C46—H46A	0.9900
C124—H124	0.9500	C46—H46B	0.9900
C125—C126	1.394 (3)	C47—H47A	0.9800
C125—H125	0.9500	C47—H47B	0.9800
C126—H126	0.9500	C47—H47C	0.9800
C151—C152	1.513 (3)	C411—C412	1.379 (3)
C151—H15A	0.9900	C411—C416	1.393 (3)
C151—H15B	0.9900	C412—C413	1.384 (3)
C152—H15C	0.9800	C412—H412	0.9500
C152—H15D	0.9800	C413—C414	1.382 (4)
C152—H15E	0.9800	C413—H413	0.9500
S24—C24	1.696 (2)	C414—C415	1.380 (4)
N21—C22	1.359 (3)	C414—H414	0.9500
N21—C211	1.419 (3)	C415—C416	1.385 (3)
N21—H21	0.8603	C415—H415	0.9500
N23—C22	1.293 (3)	C416—H416	0.9500
N23—C24	1.374 (3)	C421—C426	1.386 (3)
N25—C24	1.337 (3)	C421—C422	1.387 (3)
N25—C26	1.461 (3)	C422—C423	1.385 (4)
N25—C251	1.473 (3)	C422—H422	0.9500
C22—C221	1.495 (3)	C423—C424	1.379 (4)
C26—C27	1.515 (3)	C423—H423	0.9500
C26—H26A	0.9900	C424—C425	1.371 (4)
C26—H26B	0.9900	C424—H424	0.9500
C27—H27A	0.9800	C425—C426	1.391 (3)
C27—H27B	0.9800	C425—H425	0.9500
C27—H27C	0.9800	C426—H426	0.9500
C211—C212	1.387 (3)	C451—C452	1.499 (4)
C211—C216	1.393 (3)	C451—H45A	0.9900
C212—C213	1.380 (3)	C451—H45B	0.9900
C212—H212	0.9500	C452—H45C	0.9800
C213—C214	1.386 (4)	C452—H45D	0.9800
C213—H213	0.9500	C452—H45E	0.9800
C214—C215	1.378 (4)	S54—C54	1.696 (2)
C214—H214	0.9500	N51—C52	1.358 (3)
C215—C216	1.385 (3)	N51—C511	1.420 (3)
C215—H215	0.9500	N51—H51	0.8601
C216—H216	0.9500	N53—C52	1.291 (3)
C221—C222	1.387 (3)	N53—C54	1.375 (3)
C221—C226	1.391 (4)	N55—C54	1.340 (3)
C222—C223	1.389 (4)	N55—C56	1.467 (3)
C222—H222	0.9500	N55—C551	1.484 (3)
C223—C224	1.377 (4)	C52—C521	1.495 (3)
C223—H223	0.9500	C56—C57	1.511 (3)
C224—C225	1.365 (4)	C56—H56A	0.9900
C224—H224	0.9500	C56—H56B	0.9900
C225—C226	1.392 (3)	C57—H57A	0.9800
C225—H225	0.9500	C57—H57B	0.9800
C226—H226	0.9500	C57—H57C	0.9800
C251—C252	1.508 (3)	C511—C512	1.386 (3)
C251—H25A	0.9900	C511—C516	1.386 (3)
C251—H25B	0.9900	C512—C513	1.383 (3)
C252—H25C	0.9800	C512—H512	0.9500
C252—H25D	0.9800	C513—C514	1.387 (4)
C252—H25E	0.9800	C513—H513	0.9500
S34—C34	1.696 (2)	C514—C515	1.385 (4)
N31—C32	1.362 (3)	C514—H514	0.9500
N31—C311	1.420 (3)	C515—C516	1.386 (3)
N31—H31	0.8601	C515—H515	0.9500
N33—C32	1.295 (3)	C516—H516	0.9500
N33—C34	1.380 (3)	C521—C526	1.382 (4)
N35—C34	1.335 (3)	C521—C522	1.391 (4)
N35—C36	1.464 (3)	C522—C523	1.387 (4)
N35—C351	1.473 (3)	C522—H522	0.9500
C32—C321	1.492 (3)	C523—C524	1.368 (5)
C36—C37	1.517 (3)	C523—H523	0.9500
C36—H36A	0.9900	C524—C525	1.375 (5)
C36—H36B	0.9900	C524—H524	0.9500
C37—H37A	0.9800	C525—C526	1.390 (4)
C37—H37B	0.9800	C525—H525	0.9500
C37—H37C	0.9800	C526—H526	0.9500
C311—C312	1.384 (3)	C551—C552	1.512 (3)
C311—C316	1.389 (3)	C551—H55A	0.9900
C312—C313	1.386 (3)	C551—H55B	0.9900
C312—H312	0.9500	C552—H55C	0.9800
C313—C314	1.382 (4)	C552—H55D	0.9800
C313—H313	0.9500	C552—H55E	0.9800
			
C12—N11—C111	125.3 (2)	C316—C315—C314	120.9 (2)
C12—N11—H11	119.2	C316—C315—H315	119.5
C111—N11—H11	113.8	C314—C315—H315	119.5
C12—N13—C14	122.8 (2)	C315—C316—C311	119.7 (2)
C14—N15—C16	120.9 (2)	C315—C316—H316	120.2
C14—N15—C151	121.3 (2)	C311—C316—H316	120.2
C16—N15—C151	117.8 (2)	C326—C321—C322	119.2 (2)
N13—C12—N11	119.4 (2)	C326—C321—C32	121.1 (2)
N13—C12—C121	125.3 (2)	C322—C321—C32	119.6 (2)
N11—C12—C121	115.0 (2)	C323—C322—C321	120.3 (2)
N15—C14—N13	113.8 (2)	C323—C322—H322	119.8
N15—C14—S14	123.7 (2)	C321—C322—H322	119.8
N13—C14—S14	122.10 (18)	C324—C323—C322	120.0 (3)
N15—C16—C17	112.0 (2)	C324—C323—H323	120.0
N15—C16—H16A	109.2	C322—C323—H323	120.0
C17—C16—H16A	109.2	C325—C324—C323	120.3 (2)
N15—C16—H16B	109.2	C325—C324—H324	119.9
C17—C16—H16B	109.2	C323—C324—H324	119.9
H16A—C16—H16B	107.9	C324—C325—C326	120.5 (2)
C16—C17—H17A	109.5	C324—C325—H325	119.8
C16—C17—H17B	109.5	C326—C325—H325	119.8
H17A—C17—H17B	109.5	C325—C326—C321	119.7 (3)
C16—C17—H17C	109.5	C325—C326—H326	120.1
H17A—C17—H17C	109.5	C321—C326—H326	120.1
H17B—C17—H17C	109.5	N35—C351—C352	115.50 (19)
C112—C111—C116	119.6 (2)	N35—C351—H35A	108.4
C112—C111—N11	119.1 (2)	C352—C351—H35A	108.4
C116—C111—N11	121.4 (2)	N35—C351—H35B	108.4
C113—C112—C111	120.4 (2)	C352—C351—H35B	108.4
C113—C112—H112	119.8	H35A—C351—H35B	107.5
C111—C112—H112	119.8	C351—C352—H35C	109.5
C112—C113—C114	120.8 (3)	C351—C352—H35D	109.5
C112—C113—H113	119.6	H35C—C352—H35D	109.5
C114—C113—H113	119.6	C351—C352—H35E	109.5
C113—C114—C115	118.7 (2)	H35C—C352—H35E	109.5
C113—C114—H114	120.6	H35D—C352—H35E	109.5
C115—C114—H114	120.6	C42—N41—C411	125.1 (2)
C116—C115—C114	121.0 (2)	C42—N41—H41	118.0
C116—C115—H115	119.5	C411—N41—H41	115.4
C114—C115—H115	119.5	C42—N43—C44	123.0 (2)
C115—C116—C111	119.6 (2)	C44—N45—C46	122.2 (2)
C115—C116—H116	120.2	C44—N45—C451	121.8 (2)
C111—C116—H116	120.2	C46—N45—C451	116.0 (2)
C126—C121—C122	119.3 (2)	N43—C42—N41	120.2 (2)
C126—C121—C12	121.3 (2)	N43—C42—C421	124.2 (2)
C122—C121—C12	119.3 (2)	N41—C42—C421	115.5 (2)
C123—C122—C121	120.3 (2)	N45—C44—N43	114.8 (2)
C123—C122—H122	119.8	N45—C44—S44	123.9 (2)
C121—C122—H122	119.8	N43—C44—S44	121.09 (18)
C124—C123—C122	120.3 (3)	N45—C46—C47	111.7 (2)
C124—C123—H123	119.8	N45—C46—H46A	109.3
C122—C123—H123	119.8	C47—C46—H46A	109.3
C123—C124—C125	120.0 (2)	N45—C46—H46B	109.3
C123—C124—H124	120.0	C47—C46—H46B	109.3
C125—C124—H124	120.0	H46A—C46—H46B	107.9
C124—C125—C126	120.2 (2)	C46—C47—H47A	109.5
C124—C125—H125	119.9	C46—C47—H47B	109.5
C126—C125—H125	119.9	H47A—C47—H47B	109.5
C121—C126—C125	119.8 (2)	C46—C47—H47C	109.5
C121—C126—H126	120.1	H47A—C47—H47C	109.5
C125—C126—H126	120.1	H47B—C47—H47C	109.5
N15—C151—C152	115.1 (2)	C412—C411—C416	119.7 (2)
N15—C151—H15A	108.5	C412—C411—N41	119.3 (2)
C152—C151—H15A	108.5	C416—C411—N41	121.0 (2)
N15—C151—H15B	108.5	C411—C412—C413	120.3 (2)
C152—C151—H15B	108.5	C411—C412—H412	119.8
H15A—C151—H15B	107.5	C413—C412—H412	119.8
C151—C152—H15C	109.5	C414—C413—C412	120.0 (3)
C151—C152—H15D	109.5	C414—C413—H413	120.0
H15C—C152—H15D	109.5	C412—C413—H413	120.0
C151—C152—H15E	109.5	C415—C414—C413	119.9 (2)
H15C—C152—H15E	109.5	C415—C414—H414	120.1
H15D—C152—H15E	109.5	C413—C414—H414	120.1
C22—N21—C211	125.4 (2)	C414—C415—C416	120.4 (2)
C22—N21—H21	116.6	C414—C415—H415	119.8
C211—N21—H21	117.4	C416—C415—H415	119.8
C22—N23—C24	123.0 (2)	C415—C416—C411	119.7 (3)
C24—N25—C26	122.5 (2)	C415—C416—H416	120.2
C24—N25—C251	121.8 (2)	C411—C416—H416	120.2
C26—N25—C251	115.7 (2)	C426—C421—C422	119.2 (2)
N23—C22—N21	120.5 (2)	C426—C421—C42	121.4 (2)
N23—C22—C221	124.6 (2)	C422—C421—C42	119.3 (2)
N21—C22—C221	114.8 (2)	C423—C422—C421	120.4 (3)
N25—C24—N23	114.5 (2)	C423—C422—H422	119.8
N25—C24—S24	123.7 (2)	C421—C422—H422	119.8
N23—C24—S24	121.53 (18)	C424—C423—C422	119.8 (3)
N25—C26—C27	111.7 (2)	C424—C423—H423	120.1
N25—C26—H26A	109.3	C422—C423—H423	120.1
C27—C26—H26A	109.3	C425—C424—C423	120.3 (3)
N25—C26—H26B	109.3	C425—C424—H424	119.9
C27—C26—H26B	109.3	C423—C424—H424	119.9
H26A—C26—H26B	107.9	C424—C425—C426	120.2 (3)
C26—C27—H27A	109.5	C424—C425—H425	119.9
C26—C27—H27B	109.5	C426—C425—H425	119.9
H27A—C27—H27B	109.5	C421—C426—C425	120.0 (3)
C26—C27—H27C	109.5	C421—C426—H426	120.0
H27A—C27—H27C	109.5	C425—C426—H426	120.0
H27B—C27—H27C	109.5	N45—C451—C452	111.9 (2)
C212—C211—C216	119.8 (2)	N45—C451—H45A	109.2
C212—C211—N21	119.0 (2)	C452—C451—H45A	109.2
C216—C211—N21	121.2 (2)	N45—C451—H45B	109.2
C213—C212—C211	120.0 (2)	C452—C451—H45B	109.2
C213—C212—H212	120.0	H45A—C451—H45B	107.9
C211—C212—H212	120.0	C451—C452—H45C	109.5
C212—C213—C214	120.7 (3)	C451—C452—H45D	109.5
C212—C213—H213	119.7	H45C—C452—H45D	109.5
C214—C213—H213	119.7	C451—C452—H45E	109.5
C215—C214—C213	119.2 (2)	H45C—C452—H45E	109.5
C215—C214—H214	120.4	H45D—C452—H45E	109.5
C213—C214—H214	120.4	C52—N51—C511	125.9 (2)
C214—C215—C216	121.1 (3)	C52—N51—H51	117.5
C214—C215—H215	119.5	C511—N51—H51	116.2
C216—C215—H215	119.5	C52—N53—C54	122.0 (2)
C215—C216—C211	119.4 (3)	C54—N55—C56	121.2 (2)
C215—C216—H216	120.3	C54—N55—C551	121.4 (2)
C211—C216—H216	120.3	C56—N55—C551	117.4 (2)
C222—C221—C226	119.8 (2)	N53—C52—N51	120.4 (2)
C222—C221—C22	119.7 (2)	N53—C52—C521	125.4 (2)
C226—C221—C22	120.5 (2)	N51—C52—C521	114.0 (2)
C221—C222—C223	119.9 (3)	N55—C54—N53	113.9 (2)
C221—C222—H222	120.0	N55—C54—S54	123.4 (2)
C223—C222—H222	120.0	N53—C54—S54	122.23 (19)
C224—C223—C222	119.8 (3)	N55—C56—C57	112.0 (2)
C224—C223—H223	120.1	N55—C56—H56A	109.2
C222—C223—H223	120.1	C57—C56—H56A	109.2
C225—C224—C223	120.6 (3)	N55—C56—H56B	109.2
C225—C224—H224	119.7	C57—C56—H56B	109.2
C223—C224—H224	119.7	H56A—C56—H56B	107.9
C224—C225—C226	120.4 (3)	C56—C57—H57A	109.5
C224—C225—H225	119.8	C56—C57—H57B	109.5
C226—C225—H225	119.8	H57A—C57—H57B	109.5
C221—C226—C225	119.4 (3)	C56—C57—H57C	109.5
C221—C226—H226	120.3	H57A—C57—H57C	109.5
C225—C226—H226	120.3	H57B—C57—H57C	109.5
N25—C251—C252	111.7 (2)	C512—C511—C516	119.6 (2)
N25—C251—H25A	109.3	C512—C511—N51	118.9 (2)
C252—C251—H25A	109.3	C516—C511—N51	121.4 (2)
N25—C251—H25B	109.3	C513—C512—C511	120.2 (2)
C252—C251—H25B	109.3	C513—C512—H512	119.9
H25A—C251—H25B	107.9	C511—C512—H512	119.9
C251—C252—H25C	109.5	C512—C513—C514	120.4 (3)
C251—C252—H25D	109.5	C512—C513—H513	119.8
H25C—C252—H25D	109.5	C514—C513—H513	119.8
C251—C252—H25E	109.5	C515—C514—C513	119.2 (2)
H25C—C252—H25E	109.5	C515—C514—H514	120.4
H25D—C252—H25E	109.5	C513—C514—H514	120.4
C32—N31—C311	126.4 (2)	C514—C515—C516	120.6 (2)
C32—N31—H31	118.5	C514—C515—H515	119.7
C311—N31—H31	114.6	C516—C515—H515	119.7
C32—N33—C34	122.7 (2)	C515—C516—C511	119.9 (3)
C34—N35—C36	121.06 (19)	C515—C516—H516	120.0
C34—N35—C351	121.3 (2)	C511—C516—H516	120.0
C36—N35—C351	117.66 (19)	C526—C521—C522	120.3 (2)
N33—C32—N31	120.2 (2)	C526—C521—C52	120.6 (2)
N33—C32—C321	125.5 (2)	C522—C521—C52	119.2 (2)
N31—C32—C321	114.1 (2)	C523—C522—C521	119.7 (3)
N35—C34—N33	114.0 (2)	C523—C522—H522	120.2
N35—C34—S34	124.1 (2)	C521—C522—H522	120.2
N33—C34—S34	121.57 (18)	C524—C523—C522	119.8 (3)
N35—C36—C37	111.7 (2)	C524—C523—H523	120.1
N35—C36—H36A	109.3	C522—C523—H523	120.1
C37—C36—H36A	109.3	C523—C524—C525	120.8 (3)
N35—C36—H36B	109.3	C523—C524—H524	119.6
C37—C36—H36B	109.3	C525—C524—H524	119.6
H36A—C36—H36B	107.9	C524—C525—C526	120.1 (3)
C36—C37—H37A	109.5	C524—C525—H525	120.0
C36—C37—H37B	109.5	C526—C525—H525	120.0
H37A—C37—H37B	109.5	C521—C526—C525	119.3 (3)
C36—C37—H37C	109.5	C521—C526—H526	120.3
H37A—C37—H37C	109.5	C525—C526—H526	120.3
H37B—C37—H37C	109.5	N55—C551—C552	114.1 (2)
C312—C311—C316	119.8 (2)	N55—C551—H55A	108.7
C312—C311—N31	118.6 (2)	C552—C551—H55A	108.7
C316—C311—N31	121.6 (2)	N55—C551—H55B	108.7
C311—C312—C313	119.9 (2)	C552—C551—H55B	108.7
C311—C312—H312	120.0	H55A—C551—H55B	107.6
C313—C312—H312	120.0	C551—C552—H55C	109.5
C314—C313—C312	120.7 (3)	C551—C552—H55D	109.5
C314—C313—H313	119.7	H55C—C552—H55D	109.5
C312—C313—H313	119.7	C551—C552—H55E	109.5
C313—C314—C315	119.0 (2)	H55C—C552—H55E	109.5
C313—C314—H314	120.5	H55D—C552—H55E	109.5
C315—C314—H314	120.5		
			
C14—N13—C12—N11	172.7 (2)	C313—C314—C315—C316	−0.3 (4)
C14—N13—C12—C121	−13.1 (4)	C314—C315—C316—C311	1.0 (4)
C111—N11—C12—N13	2.7 (3)	C312—C311—C316—C315	−1.2 (4)
C111—N11—C12—C121	−172.0 (2)	N31—C311—C316—C315	−178.8 (2)
C16—N15—C14—N13	−178.8 (2)	N33—C32—C321—C326	137.4 (3)
C151—N15—C14—N13	1.3 (3)	N31—C32—C321—C326	−47.7 (3)
C16—N15—C14—S14	8.0 (3)	N33—C32—C321—C322	−45.7 (3)
C151—N15—C14—S14	−171.87 (17)	N31—C32—C321—C322	129.3 (2)
C12—N13—C14—N15	127.4 (2)	C326—C321—C322—C323	0.4 (4)
C12—N13—C14—S14	−59.2 (3)	C32—C321—C322—C323	−176.6 (2)
C14—N15—C16—C17	84.2 (3)	C321—C322—C323—C324	−0.7 (4)
C151—N15—C16—C17	−96.0 (3)	C322—C323—C324—C325	0.2 (4)
C12—N11—C111—C112	131.9 (3)	C323—C324—C325—C326	0.7 (4)
C12—N11—C111—C116	−48.4 (3)	C324—C325—C326—C321	−0.9 (4)
C116—C111—C112—C113	0.9 (4)	C322—C321—C326—C325	0.4 (4)
N11—C111—C112—C113	−179.4 (2)	C32—C321—C326—C325	177.3 (2)
C111—C112—C113—C114	−0.4 (4)	C34—N35—C351—C352	−77.3 (3)
C112—C113—C114—C115	−0.2 (4)	C36—N35—C351—C352	102.0 (3)
C113—C114—C115—C116	0.4 (4)	C44—N43—C42—N41	−169.5 (2)
C114—C115—C116—C111	0.1 (4)	C44—N43—C42—C421	14.3 (4)
C112—C111—C116—C115	−0.7 (4)	C411—N41—C42—N43	0.7 (4)
N11—C111—C116—C115	179.6 (2)	C411—N41—C42—C421	177.1 (2)
N13—C12—C121—C126	134.9 (3)	C46—N45—C44—N43	−173.9 (2)
N11—C12—C121—C126	−50.7 (3)	C451—N45—C44—N43	7.3 (3)
N13—C12—C121—C122	−47.3 (3)	C46—N45—C44—S44	1.1 (3)
N11—C12—C121—C122	127.1 (2)	C451—N45—C44—S44	−177.82 (17)
C126—C121—C122—C123	0.6 (4)	C42—N43—C44—N45	−119.8 (2)
C12—C121—C122—C123	−177.1 (2)	C42—N43—C44—S44	65.1 (3)
C121—C122—C123—C124	−0.8 (4)	C44—N45—C46—C47	−91.2 (3)
C122—C123—C124—C125	0.2 (4)	C451—N45—C46—C47	87.7 (3)
C123—C124—C125—C126	0.6 (4)	C42—N41—C411—C412	−133.9 (3)
C122—C121—C126—C125	0.1 (4)	C42—N41—C411—C416	47.9 (3)
C12—C121—C126—C125	177.8 (2)	C416—C411—C412—C413	−1.0 (4)
C124—C125—C126—C121	−0.7 (4)	N41—C411—C412—C413	−179.2 (2)
C14—N15—C151—C152	−78.0 (3)	C411—C412—C413—C414	−0.1 (4)
C16—N15—C151—C152	102.2 (3)	C412—C413—C414—C415	1.2 (4)
C24—N23—C22—N21	−167.5 (2)	C413—C414—C415—C416	−1.1 (4)
C24—N23—C22—C221	16.4 (4)	C414—C415—C416—C411	0.0 (4)
C211—N21—C22—N23	6.7 (4)	C412—C411—C416—C415	1.1 (4)
C211—N21—C22—C221	−176.8 (2)	N41—C411—C416—C415	179.2 (2)
C26—N25—C24—N23	−172.16 (19)	N43—C42—C421—C426	−130.8 (3)
C251—N25—C24—N23	7.2 (3)	N41—C42—C421—C426	52.9 (3)
C26—N25—C24—S24	2.1 (3)	N43—C42—C421—C422	49.1 (3)
C251—N25—C24—S24	−178.55 (17)	N41—C42—C421—C422	−127.2 (3)
C22—N23—C24—N25	−127.5 (2)	C426—C421—C422—C423	−0.1 (4)
C22—N23—C24—S24	58.1 (3)	C42—C421—C422—C423	180.0 (2)
C24—N25—C26—C27	−93.4 (3)	C421—C422—C423—C424	0.8 (4)
C251—N25—C26—C27	87.2 (2)	C422—C423—C424—C425	−0.8 (4)
C22—N21—C211—C212	−137.0 (3)	C423—C424—C425—C426	0.1 (4)
C22—N21—C211—C216	46.3 (3)	C422—C421—C426—C425	−0.5 (4)
C216—C211—C212—C213	−0.4 (4)	C42—C421—C426—C425	179.4 (2)
N21—C211—C212—C213	−177.1 (2)	C424—C425—C426—C421	0.5 (4)
C211—C212—C213—C214	0.3 (4)	C44—N45—C451—C452	−86.6 (3)
C212—C213—C214—C215	0.0 (4)	C46—N45—C451—C452	94.5 (3)
C213—C214—C215—C216	−0.2 (4)	C54—N53—C52—N51	170.8 (2)
C214—C215—C216—C211	0.2 (4)	C54—N53—C52—C521	−13.9 (4)
C212—C211—C216—C215	0.2 (4)	C511—N51—C52—N53	−3.8 (4)
N21—C211—C216—C215	176.8 (2)	C511—N51—C52—C521	−179.6 (2)
N23—C22—C221—C222	48.2 (4)	C56—N55—C54—N53	−178.78 (19)
N21—C22—C221—C222	−128.1 (3)	C551—N55—C54—N53	−0.3 (3)
N23—C22—C221—C226	−133.3 (3)	C56—N55—C54—S54	8.4 (3)
N21—C22—C221—C226	50.4 (3)	C551—N55—C54—S54	−173.14 (17)
C226—C221—C222—C223	0.1 (4)	C52—N53—C54—N55	132.6 (2)
C22—C221—C222—C223	178.6 (2)	C52—N53—C54—S54	−54.5 (3)
C221—C222—C223—C224	0.8 (4)	C54—N55—C56—C57	83.7 (3)
C222—C223—C224—C225	−0.8 (5)	C551—N55—C56—C57	−94.8 (3)
C223—C224—C225—C226	−0.3 (5)	C52—N51—C511—C512	138.6 (3)
C222—C221—C226—C225	−1.1 (4)	C52—N51—C511—C516	−44.5 (4)
C22—C221—C226—C225	−179.7 (2)	C516—C511—C512—C513	1.0 (4)
C224—C225—C226—C221	1.2 (4)	N51—C511—C512—C513	178.0 (2)
C24—N25—C251—C252	−88.3 (3)	C511—C512—C513—C514	−0.3 (4)
C26—N25—C251—C252	91.1 (3)	C512—C513—C514—C515	−0.5 (4)
C34—N33—C32—N31	170.6 (2)	C513—C514—C515—C516	0.4 (4)
C34—N33—C32—C321	−14.7 (4)	C514—C515—C516—C511	0.3 (4)
C311—N31—C32—N33	−3.1 (4)	C512—C511—C516—C515	−1.1 (4)
C311—N31—C32—C321	−178.4 (2)	N51—C511—C516—C515	−178.0 (2)
C36—N35—C34—N33	−179.83 (19)	N53—C52—C521—C526	129.5 (3)
C351—N35—C34—N33	−0.6 (3)	N51—C52—C521—C526	−55.0 (3)
C36—N35—C34—S34	6.2 (3)	N53—C52—C521—C522	−50.4 (4)
C351—N35—C34—S34	−174.57 (16)	N51—C52—C521—C522	125.1 (3)
C32—N33—C34—N35	128.7 (2)	C526—C521—C522—C523	0.9 (4)
C32—N33—C34—S34	−57.2 (3)	C52—C521—C522—C523	−179.3 (2)
C34—N35—C36—C37	86.7 (3)	C521—C522—C523—C524	−1.0 (4)
C351—N35—C36—C37	−92.6 (2)	C522—C523—C524—C525	0.5 (5)
C32—N31—C311—C312	137.7 (3)	C523—C524—C525—C526	0.0 (5)
C32—N31—C311—C316	−44.7 (3)	C522—C521—C526—C525	−0.4 (4)
C316—C311—C312—C313	0.8 (4)	C52—C521—C526—C525	179.8 (2)
N31—C311—C312—C313	178.4 (2)	C524—C525—C526—C521	−0.1 (4)
C311—C312—C313—C314	0.0 (4)	C54—N55—C551—C552	−77.8 (3)
C312—C313—C314—C315	−0.2 (4)	C56—N55—C551—C552	100.7 (3)

### Hydrogen-bond geometry (Å, °)

**Table:**

Cg2, Cg4, Cg5, Cg8 and Cg10 are the centroids of the C121–C126, C221–C226, C321–C326, C421–C426 and C521–C526 rings, respectively.

**Table:**

*D*—H···*A*	*D*—H	H···*A*	*D*···*A*	*D*—H···*A*
N11—H11···S24	0.86	2.62	3.440 (2)	159
N21—H21···S34	0.86	2.60	3.407 (2)	157
N31—H31···S44	0.86	2.64	3.459 (2)	161
N41—H41···S54	0.86	2.62	3.416 (2)	155
N51—H51···S14^i^	0.86	2.62	3.431 (2)	157
C26—H26B···Cg2	0.99	2.91	3.594 (4)	127
C36—H36A···Cg4	0.99	2.86	3.575 (4)	130
C46—H46B···Cg6	0.99	2.84	3.549 (4)	129
C56—H56A···Cg8	0.99	2.89	3.566 (4)	126
C16—H16A···Cg10^ii^	0.99	2.86	3.552 (4)	128

Symmetry codes: (i) *x*+2, −*y*+1/2, *z*+3/2; (ii) *x*−2, −*y*−1/2, *z*−5/2.
